# Rosacea: Molecular Mechanisms and Management of a Chronic Cutaneous Inflammatory Condition

**DOI:** 10.3390/ijms17091562

**Published:** 2016-09-15

**Authors:** Yu Ri Woo, Ji Hong Lim, Dae Ho Cho, Hyun Jeong Park

**Affiliations:** 1 Department of Dermatology, Yeouido St. Mary’s Hospital, College of Medicine, The Catholic University of Korea, Seoul 07345, Korea; w1206@naver.com (Y.R.W.); redtor@hanmail.net (J.H.L.); 2 Department of Life Science, Sookmyung Women’s University, Seoul 04310, Korea; cdhkor@sookmyung.ac.kr

**Keywords:** rosacea, genetic, triggering factor, immune defect, inflammation, neurovascular dysregulation

## Abstract

Rosacea is a chronic cutaneous inflammatory disease that affects the facial skin. Clinically, rosacea can be categorized into papulopustular, erythematotelangiectatic, ocular, and phymatous rosacea. However, the phenotypic presentations of rosacea are more heterogeneous. Although the pathophysiology of rosacea remains to be elucidated, immunologic alterations and neurovascular dysregulation are thought to have important roles in initiating and strengthening the clinical manifestations of rosacea. In this article, we present the possible molecular mechanisms of rosacea based on recent laboratory and clinical studies. We describe the genetic predisposition for rosacea along with its associated diseases, triggering factors, and suggested management options in detail based on the underlying molecular biology. Understanding the molecular pathomechanisms of rosacea will likely aid toward better comprehending its complex pathogenesis.

## 1. Introduction

Rosacea is a common chronic inflammatory skin condition characterized by erythema, papules, telangiectasia, edema, pustules, or a combination of these symptoms [1]. Most of the skin lesions of rosacea generally occur on the central face, such as the cheeks, forehead, chin, and nose [2]. Patients also suffer from cutaneous symptoms, such as facial flushing, stinging, pain or burning sensations. The clinical subtypes of rosacea are erythematotelangiectatic, papulopustular, phymatous, and ocular [3]. However, in practice, various subtypes can be manifested in one patient, and the clinical manifestations of rosacea are more diverse.

Owing to its heterogeneity, the results from epidemiologic studies regarding the incidence and prevalence of rosacea are generally varied. The incidence rate of rosacea has been recently identified as 1.65 per 1000 persons per year [4]. The prevalence of rosacea is usually stated to develop after the age of 30 [5]. Although the reported data have been obtained mainly based on adults, a prevalence of rosacea in childhood also exists, although the exact age of onset of rosacea remains to be explored [6,7]. Furthermore, whereas most patients with rosacea are fair-skinned individuals [2], recent reports indicate that rosacea can develop in other skin phototypes as well, including in Asian and African populations [8,9].

**Figure 1:**
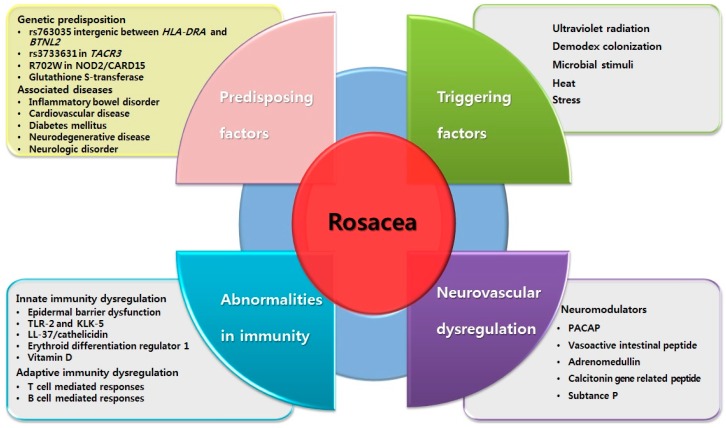
Schematic view of the factors known to contribute to the molecular mechanisms of rosacea.

Numerous factors are known to contribute to the molecular mechanisms of rosacea (Figure 1). Among these, abnormalities in the innate immune system and neurovascular dysregulation have been considered to be primarily implicated in the pathophysiology of rosacea [10]. The skin is an important organ for maintaining the homeostasis of innate immunity, which represents a type of host defense mechanism against invading pathogens. In addition to the physical barrier of the skin, the innate immune system is regulated by various factors, including associated molecules such as antimicrobial peptides (AMPs) and pattern recognition receptors (PRRs). Several researchers have observed an altered innate immune system in patients with rosacea [11,12]. Abnormalities of innate immunity can increase the skin’s susceptibility to the external environment, which might represent an influential factor in initiating or aggravating rosacea. Additionally, studies have also recently been conducted to identify possible abnormalities in adaptive immunity in rosacea, which might contribute to further inflammatory responses in rosacea [13,14].

Recent advances in our understanding of the etiology of rosacea also include predisposing factors such as genetic susceptibility as well as its potential association with other chronic disorders [15,16]. In addition, various triggering factors including demodex colonization, microbial stimuli, ultraviolet (UV) radiation, heat, and stress are considered to be related to the initiation or aggravation of rosacea.

Here, we review the molecular mechanisms of rosacea, focusing on recent advances in genetic predisposing factors along with possible associated diseases. This review also presents the triggering factors for rosacea in detail, based on their supposed mechanisms. In addition, we describe the neurovascular dysregulation in rosacea and present the immune system abnormalities by classifying them into innate and adaptive immune responses (Figure 1). Finally, we discuss the therapeutic options for rosacea based on the underlying molecular mechanisms of action.

## 2. Predisposing Factors for Rosacea

### 2.1. Genetics

Data regarding the inheritance of rosacea are scarce. Patients with rosacea have a markedly increased tendency to exhibit a positive family history than do control groups [17]. Furthermore, owing to its higher prevalence among Northern Europeans, a genetic predisposition toward developing rosacea has been hypothesized; however, the specific genes related to this association have not yet been identified [3].

In 2007, a case of rosacea in monozygotic twins was reported, which first suggested a possible genetic role in its pathophysiology [18]. Additionally, a recent cohort study of twins with rosacea found a higher correlation of National Rosacea Society clinical scores between monozygous than between heterozygous twins [15]. This cohort study also indicated that approximately half of the factors affecting the pathophysiology of rosacea were genetic, whereas the remainders were environmental, such as smoking, alcohol consumption, skin cancer history, and age [15].

Another genetic analysis demonstrated the potential relevance of a polymorphism in glutathione S-transferase (GST) in rosacea [19], wherein the GSTT1 and GSTM1 null genotypes were found to be significantly associated with an increased disease risk [19]. Because GST encodes an enzyme required for catalyzing reactive oxygen species (ROS), polymorphism in GST might lead to increased oxidative stress and affect the pathogenic mechanisms in rosacea.

Furthermore, a recent genome-wide association study (GWAS) by Chang et al. [20] identified two single-nucleotide polymorphisms (SNPs), rs763035 and rs111314066, in a large population of European individuals with rosacea. However, only rs763035, which is intergenic between the butyrophilin-like 2 (*BRNL2*) and human leukocyte antigen (*HLA*)-*DRA* loci, was confirmed in the replication group [20]. An exploratory immunohistochemical study using antibodies against HLA-DRA showed strong staining at sites of perifollicular inflammatory infiltrates, epidermal Langerhans cells, and endothelial cells. In comparison, staining with a BRNL2 antibody revealed diffuse expression in keratinocytes, perifollicular inflammatory infiltrates, and endothelial cells of papulopustular rosacea, which support the concept that the genes associated with rs763035 are expressed in rosacea skin samples [20]. In addition, they reported that three major histocompatibility complex (MHC) class II alleles including HLA-DRB1*, HLA-DQB1*, and HLA-DQA1* are also associated with rosacea [20].

Finally, the R702W polymorphism in NOD2/CARD15 was observed in a patient with granulomatous rosacea [21]. NOD2/CARD15 is a member of the N-terminal caspase recruitment protein domain family and participates in the inflammatory response mediated by toll like receptor (TLR) [21]. In turn, a genetic predisposition to carrying the rs3733631 polymorphic variant in the tachykinin receptor gene *TACR3* was also observed in patients with rosacea [22]; notably, this is near the *TLR2* gene locus at 4q25 [22]. It is therefore reasonable to suppose that the polymorphism in *TACR3* might be involved in the development of rosacea by enhancing the expression of TLR2.

### 2.2. Associated Diseases

The *HLA-DRA* locus is associated with rosacea as well as with other inflammation-associated disorders, such as inflammatory bowel diseases including ulcerative colitis, Crohn’s disease, and celiac disease [20,23,24]. A recent population-based study of rosacea also found this association [25,26]. Moreover, Spoendlin et al. [25] found that an increased risk of rosacea was observed particularly during the period of increased gastrointestinal tract inflammation. Thus, the overlap in the genetic relevance of *HLA-DRA* between rosacea and inflammatory bowel diseases might imply a potential link between these disorders

Patients with rosacea have a higher risk of cardiovascular comorbidities including hypertension, dyslipidemia, and coronary artery disease than that seen in controls [27,28,29]. Rosacea severity was also found to be dependent on the presence of cardiovascular comorbidities [29]. One explanation for this association might be the level of the high-density lipoprotein paraoxonase-1, which was decreased in patients with rosacea and dyslipidemia [30,31]. In addition, the association between cardiovascular diseases and rosacea might also be explained by enhanced expression of the cathelicidin, which has been observed both in the course of atherosclerosis and rosacea [27].

The GWAS by Chang et al. [20] also revealed that patients with rosacea shared a genetic locus with type 1 diabetes mellitus; this association was further confirmed by a population-based study as well [26]. A recent study also revealed the presence of insulin resistance in rosacea, demonstrating significantly higher fasting blood glucose levels in patients with rosacea than in controls [32]. As increased glucose intolerance along with dyslipidemia and hypertension are the factors that comprise metabolic syndrome, this association in rosacea might be related to cathelicidin, oxidative stress and endoplasmic reticulum (ER) stress, which are implicated in the pathogenesis of both rosacea and metabolic disorders [32]. However, the advanced state of diabetes mellitus, which is associated with longer disease duration or high hemoglobin A1_c_ levels, has conversely been recently associated with a decreased risk of rosacea [33]. Thus, the underlying mechanism involved in this paradoxical association remains to be clarified.

Patients with rosacea have a significantly increased risk of neurologic disorders such as migraine, depression, complex regional pain syndrome, and glioma [34,35]. Enhanced expression of matrix metalloproteinase (MMP) is observed in these neurologic disorders as well as in rosacea, which might explain the possible shared pathogenic mechanisms between these conditions [35].

Recently, an increased interest has been shown in the potential associations between neurodegenerative diseases and rosacea. For example, a nationwide cohort study from Denmark explored the relationship between rosacea and neurodegenerative diseases such as Parkinson’s disease [36]. MMPs are believed to be associated with the neurodegenerative diseases and an increased expression of MMP-1 and MMP-9 has also been observed in rosacea [37]. In addition, another Danish study found that rosacea was significantly associated with dementia, especially Alzheimer disease [38]. AMPs, MMP, and inflammatory cascades, which have a shared impact on both rosacea and Alzheimer disease, are considered to be involved in the underlying mechanism. Together, these findings suggest that a pathogenic link might therefore exist between rosacea and neurodegenerative diseases

## 3. Triggering Factors for Rosacea

### 3.1. Ultraviolet (UV) Radiation

Many chronic inflammatory cutaneous diseases, such as rosacea and psoriasis, are known to be associated with dermal remodeling after UV irradiation. Chronic ultraviolet A (UVA) irradiation can induce the overexpression of MMP-1, which is associated with the dermal collagen degeneration seen in rosacea [39]. Furthermore, solar elastosis, which is one of the typical histopathologic findings in rosacea, has been observed after chronic UV radiation [40]. Additionally, in cultured keratinocytes, UVB upregulates the mRNA and protein expression of vascular endothelial growth factor (VEGF), a potent angiogenic factor [41]. Exposure of murine skin to UVB resulted in the production of angiogenic molecules and in fibroblast growth factor 2 (FGF2) from keratinocytes [42]. However, the particular UVA or UVB wavelength that is most influential in triggering rosacea has not yet been determined [43].

UV radiation also contributes to producing ROS. The level of ROS is higher in patients with rosacea than healthy controls [44]. ROS can promote the activation of the inflammasome, proinflammatory cytokines, and inflammatory mediators produced by keratinocytes and fibroblasts; therefore ROS can further propagate the inflammatory responses common in rosacea [45]. Furthermore, an accumulation of serum peroxide and decreased tissue superoxide dismutase activities were observed in patients with rosacea after UV irradiation, implying that imbalances between oxidant and antioxidant pathways might be involved in rosacea [46,47].

ER stress can also be induced by UV irradiation. UV-induced ER stress has been shown to increase the level of CCAAT-enhancer-binding proteins (C/EBP) homologous protein through the protein kinase R-like ER kinase (PERK) pathway [48]. Activation of PERK results in the increased expression of activating transcription factor 4, which is a major ER stressor [48]. In turn, upregulation of activating transcription factor 4 is associated with the activation of TLR2 [48]. Therefore, UV-mediated ER stress could be associated with triggering the innate immune responses in rosacea.

This potential connection is further supported by a recent study of human keratinocytes wherein the expression of myeloid differentiation factor 88 (MyD88), an adaptor molecule for TLR signaling, was increased after UV irradiation [49]. As TLR/MyD88 signaling is necessary for proinflammatory cytokine activation, a link might exist between UV radiation and TLR/MyD88 signaling in the inflammatory cascades of rosacea as well.

Together, these findings suggest that remodeling of the vasculature and dermal matrix by VEGF, FGF2, and MMP-1 and the production of ROS and ER stressors after UV radiation provide a logical framework explaining UV radiation as a triggering factor for rosacea.

### 3.2. Demodex Colonization

*Demodex folliculorum* is a species of facial mite readily found in the pilosebaceous units of humans. Individuals with rosacea exhibit a markedly increased density of demodex on their skin compared to controls in studies with skin surface biopsy specimen [50,51]. In addition, a higher population density of demodex was also observed in patients with rosacea in quantification studies using a polymerase chain reaction amplification method and a reflectance confocal microscopy [52,53]. Furthermore, a reduction in the density of demodex mites was observed after treatment, which was correlated with clinical improvement as measured by skin surface biopsy [54]. This reduction was also observed by reflectance confocal microscopy [55].

The increased number of demodex mite exoskeletons might themselves act as pathogen-associated molecular patterns, with the chitin released from demodex mites potentially prompting inflammatory responses from keratinocytes through a TLR-2 pathway [56]. A recent study has shown that skin samples with higher demodex densities exhibited enhanced expression of genes for interleukin (IL)-8, IL-1β, tumor necrosis factor (TNF)-α, cyclooxygenase-1, and the inflammasome [52]. Consistent with this, *Demodex folliculorum* has been shown to facilitate the activation of the NLRP3 inflammasome, which subsequently associates with caspase-1 and results in the release of the proinflammatory cytokine IL-1β [57].

Furthermore, the inflammatory reactions might also be aggravated when dead mites release their resident bacteria, which would likely further induce the chemotaxis of neutrophils [58]. Chemotactic factors such as IL-8 and TNF-α in turn attract more neutrophils to the tissue and thereby aggravate the inflammatory reaction [59]. Moreover, activated neutrophils induce the release of cathelicidin and MMP-9, which cause further tissue damage.

Researchers have suggested various mechanisms to explain the association between demodex mites and rosacea. For example, a genetic susceptibility effected by HLA-Cw2 and HLA-Cw4 might alter the local immune reaction pattern to demodex mites and promote their proliferation and survival [60], suggested by the finding of Akilov et al. [16] that these haplotypes were associated with increased demodex mite density. The frequent occurrence of demodecidosis in patients with immunodeficiency and vascular insufficiency suggests that an abnormal vascularized surface as well as a compromised patient immune status along with genetic predisposition might be suspected as factors promoting the proliferation of demodex in rosacea [61,62,63]. Thus, demodex proliferation facilitated by compromised tissue in rosacea may lead to exacerbation of the pathologic condition through enhanced inflammation mediated by host response to both the mites themselves and their harbored microorganisms.

### 3.3. Microbial Stimuli

Microbial stimuli for exacerbating rosacea have been hypothesized; however, the exact pathomechanisms are unclear. *Staphylococcus epidermidis* represents a common species in the normal microflora of the skin although it is sometimes considered as an accidental pathogen [64]. Increased growth of pure *S. epidermidis* was observed from pustules of patients with rosacea when compared with that from the surrounding skin [65]. In addition, when *S. epidermidis* from patients with rosacea were incubated, the production of protein profiles varied with increasing temperature [66] suggesting that the temperature might influence the behavior of *S. epidermidis* in rosacea.

*Bacillus oleronius* is a gram-negative bacterium whose role has emerged since it was first cultured from demodex mites of patients with rosacea [67]. The majority of patients with rosacea demonstrated the serum reactivity to 62-kDa and 83-kDa proteins from *B. oleronius* [67], suggesting that it might be implicated in rosacea. Given that these proteins have been shown to stimulate TLR-2, this may explain the potential of this microbe as the immunogenic antigen involved in the TLR-2-mediated inflammatory cascade found in patients with rosacea [67]. In addition, corneal epithelial cells exposed to *Bacillus* proteins were found to upregulate the expression of many inflammatory mediators including IL-6, IL-8, TNF-α, MMP-3, and MMP-9 [59]. Moreover, exposure of neutrophils to *Bacillus* proteins resulted in further neutrophil activation via inositol 1,4,5-triphosphate, and elevated production of IL-1β and IL-6 [58]. Therefore, *Bacillus* proteins could be related to the pathogenesis of rosacea by inducing chemotaxis and the production of many proinflammatory mediators [68,69].

The prevalence of *Helicobacter pylori* infection was also found to be higher in patients with rosacea than in controls [70]. However, other studies have failed to demonstrate a relationship between *H. pylori* and rosacea [71]. Increased levels of ROS and nitric oxide (NO) by *H. pylori* were found to be associated with vasodilatation and inflammation in rosacea [72,73,74]. Additionally, cytotoxin-associated gene A, known as a *H. pylori* virulence factor, facilitated the secretion of proinflammatory cytokines in the gastric epithelium, and the presence of cytotoxin-associated gene A antibodies was observed in patients with rosacea [75]. Therefore, further studies are required to verify this association.

### 3.4. Heat

Ozkol et al. [75] reported that frequently exposed to heat from using a tandoor oven exhibited a significantly higher incidence of rosacea than control subjects. They concluded that heat transmitted from the oven might represent the aggravating factor for rosacea because it likely induced capillary vasodilation. Consistent with this supposition, increased cutaneous blood flow was observed in patients with papulopustular rosacea during their exposure to local heat when assessed by laser Doppler imaging [76]. The underlying mechanisms for increased cutaneous blood flow during local heat might be explained by the initial fast-responding vasodilation mediated by axon reflexes and the secondary slow-responding vasodilation associated with NO [77]. In addition, during exposure to heat, patients with rosacea exhibited the altered control of vascular endothelial cells, which increases the skin blood flow [78].

Furthermore, heat can activate the transient receptor potential (TRP) cation channel subfamily V member 1 (TRPV1) and ankyrin 1, which are located on both neuronal and non-neuronal cells, were shown to be activated by heat in patients with rosacea [79]. Enhanced and sustained expression of TRPVs results in flushing, vasodysregulation, and neurogenic leukocyte inflammation in patients with rosacea [10]. In a recent study, dermal immunostaining for these proteins demonstrated positive staining in erythematotelangiectatic and papulopustular rosacea [79], and *TRPV1* and *TRPV2* gene expression was elevated in the erythematotelangiectatic and papulopustular types, respectively [79]. Thus, although each TRPV acts through its own mechanisms in rosacea, they all appear to be activated by heat and have potential roles in the clinical symptoms of rosacea including flushing, telangiectasia, and inflammation.

### 3.5. Stress

Muller et al. [80] reported that mental stress leads to an increase in skin sympathetic nerve activity (SSNA). SSNA is involved in vasodilatory activities and has been shown to elucidate intermittent vasodilatation on the skin [81]. SSNA hyperresponsiveness after mental stress was observed in the supraorbital skin of patients with rosacea [82]. Such exaggerated sympathetic responses might trigger the symptoms of rosacea and also cause local inflammation and neurovascular dysregulation in these patients.

In addition, cortisol releasing hormone (CRH), well-known as a master stress hormone, interacts with CRH receptors type 1 and 2, and CRH-binding protein [83]. Through CRH receptors type 1, CRH is able to induce the degranulation of mast cells and release many vasodilatory mediators, such as histamine and NO [84]. CRH is also able to mediate the production of proinflammatory cytokines including IL-6, IL-8, and IL-18, which regulate mitogen-activated protein kinase and nuclear factor κ-light-chain-enhancers of activated B cells (NF-κB) and lead to facial erythema. Furthermore, CRH receptors type 2 is expressed mainly in blood vessels and acts as a direct vasodilator, which can be observed in the skin of rosacea [85]. Nevertheless, the precise role of stress in rosacea remains unclear and requires further study.

## 4. Abnormalities in Immune System

### 4.1. Dysregulation in Innate Immunity

#### 4.1.1. Epidermal Barrier Disruption

The role of the skin as a physical barrier is important to the innate immune system. In various chronic inflammatory cutaneous diseases, such as rosacea, atopic dermatitis, and psoriasis, disturbances in the epidermal barrier are thought to represent the major contributing factor in pathogenesis [86,87]. One explanation for the observed epidermal barrier disruption is an increase in transepidermal water loss in the skin of patients with rosacea [88]. Reduced epidermal hydration levels have been observed in skin with papulopustular rosacea and the centrofacial epidermis of patients was found to be more alkaline than that in healthy controls [89]. Given that protease activity increases at an alkaline pH, the abnormal epidermal barrier function in rosacea might be associated with enhanced activation of epidermal proteases, especially kallikrein (KLK)-5.

Rosacea is also associated with sensitive skin, which usually accompanies other facial dermatoses with epidermal barrier disruption [90]. The condition of sensitive skin results in decreased tolerance to commonly used topical agents including soaps, hot water, retinoic acid, and lactic acid, which can contribute to the worsening of burning sensation and stinging in rosacea [90]. Indeed, reactivity of lactic acid stinging test was increased in patients with rosacea [88]. In addition, low irritant threshold to sodium lauryl sulfate was also observed, which demonstrating the impaired skin barrier and contact hypersensitivity in rosacea [91].

Furthermore, demodex mites themselves may lead to the disruption of the skin barrier [54]. As demodex is known to feed on epithelial cells, they can directly cause breaches in the skin. Such permanent micro-abrasion of the skin by demodex might lead to hypersensitivity in rosacea [54]. Therefore, future treatments for rosacea should include a focus on restoring skin barrier function.

#### 4.1.2. TLR-2 and KLK-5

The cells of the innate immune system in the skin express PRRs that recognize damage-associated molecular patterns or pathogen-associated molecular patterns. TLRs comprise a major subset of PRRs; among these, the expression of TLR-2 is increased in inflammatory skin disorders such as acne and rosacea [91,92]. However, this increased TLR-2 expression cannot be observed in patients with other chronic inflammatory cutaneous diseases, such as psoriasis and atopic dermatitis, implying its special role in rosacea [91].

When TLR-2 is activated by external stimuli or triggering factors for rosacea, keratinocytes produce proinflammatory cytokines and chemokines. For example, skin samples from patients with rosacea exhibit increased gene expression for proinflammatory cytokines such as IL-8, IL-1β, and TNF-α [52]. IL-8 leads to the chemotaxis of neutrophils in the skin and consequently affects the release of proteases including cathepsin G, elastase, and protease-3 [93]. Furthermore, IL-1β and TNF-α have an additional role as angiogenic factors VEGF; thus, these cytokines might explain the vascular hyper-reactivity seen in rosacea [94].

TLR-2 is also able to facilitate the activation of the NLRP3 inflammasome, which mediates IL-1β release and further inflammatory reactions [95]. These observations indicate that the NLRP3 inflammasome might play a pivotal role in IL-1β expression, which is elevated in rosacea and mediates inflammatory responses therein.

TLR-2 is further known to increase the expression of the serine protease KLK-5, which was previously identified as a stratum corneum tryptic enzyme based on its function and as the enzyme responsible for processing cathelicidin because it controls the enzymatic processing of hCAP18 into its active form, LL-37 [96]. Notably, the lesional skin of patients with rosacea was shown to express more KLK-5 than the skin of healthy controls [12].

In addition, Yamasaki et al. [91] revealed that the increased capacity for releasing KLK-5 is calcium dependent; the ligand for TLR-2 triggers an influx of calcium, which in turn induces the increased release of KLK-5. In this process, calcium functions as the stimulus for transcription of KLK-5, with TLR-2 regulating the functional release of KLK-5 [91]. In turn, KLK-5 protein activity can be regulated by serine protease inhibitors of Kazal-type (SPINKs) [93]. Among these, SPINK6 was identified in human skin as a specific inhibitor of KLKs [93]. However, its putative role in rosacea should be further explored.

KLK-5 is also mediated by MMPs. Specifically, the activation of KLK-5 occurs after the cleavage of its proenzyme form by MMP-9 [3]. Notably, enhanced expression of MMP-2 and MMP-9 was observed in the skin of patients with rosacea [97]. Such increased expression of MMPs has potential implications for the solar-mediated degeneration that is observed in rosacea [98]. In addition, MMP-mediated initiation of KLK-5 activation leads to further activation of LL-37, the processed form of cathelicidin.

#### 4.1.3. LL-37/Cathelicidin

AMPs have an important effect on many inflammatory skin disorders, such as atopic dermatitis acne, psoriasis, and rosacea by serving first defense mechanism against various organisms, and providing an initiating bridge towards adaptive immune system [99]. Patients with rosacea exhibit increased expression of cathelicidin in the epidermis [99]. In humans, only a single cathelicidin gene, cathelicidin AMP (*CAMP*), has been identified [100]. The propeptide of CAMP, termed LL-37, is mainly composed of N- and C-terminal peptides that effect its antimicrobial action [101].

A possible role for LL-37 in rosacea has been suggested by several recent studies. Notably, patients with rosacea were shown to express higher levels of LL-37 than controls [12,102]; furthermore, the higher molecular weight forms of LL-37 in particular were expressed in the epidermis of such patients [12,103]. CAMP RNA and protein expression was also higher in the skin of patients with rosacea than in healthy controls [104]. In addition, the functional relevance of these observations were demonstrated using a murine model, whereupon injection of the LL-37 peptide into BALB/c mice led to the exhibition of clinical features of rosacea, such as telangiectasia, erythema, and inflammation [105].

Along with its antimicrobial activity, LL-37 exhibits multiple functions that influence various processes including immune modulation, neutrophil chemotaxis, and the induction of cytokine and chemokine release from mast cells [106,107]. LL-37 is a compelling chemoattractant for mast cells and subsequently regulates the antimicrobial activity of these cells [108]. Unlike the response in wild-type mice, injection of LL-37 into the skin of mast cell-deficient B6.Cg-Kit W-sh HNihrJaeBsmJ (KitW-sh) mice did not induce rosacea-like clinical features such as erythema and telangiectasia [109], suggesting the importance of mast cells to the development of rosacea phenotypes. Furthermore, following LL-37 injection in wild-type animals, murine mast cells were able to enhance the expression of IL-1, IL-6, and MMP-9, which have been known to augment inflammatory responses in rosacea [110]. In addition, LL-37 affects angiogenesis through enhancing the proliferation of endothelial cells [104]. Thus, among the various functions of LL-37, the vasoactive and proinflammatory capacities of this peptide might distinctively contribute to the pathophysiology of rosacea as opposed to other cutaneous inflammatory disorders.

Finally, Kim et al. [111] observed an association between LL-37 and protease-activated receptor 2 (PAR-2). On immunohistochemical staining, skin samples from patients with rosacea demonstrated higher levels of both cathelicidin and PAR-2 expression than those from healthy controls [111]. Furthermore, in an in vitro study, increased cathelicidin mRNA expression was observed after treatment with PAR-2 activating peptides in keratinocytes [111]. These observations reflect the possible implication that increased expression of LL-37 might be mediated by activation of PAR-2 in rosacea.

#### 4.1.4. Vitamin D

In many cutaneous inflammatory skin diseases such as atopic dermatitis and chronic urticaria, patients showed significantly lower levels of vitamin D than the healthy controls [112,113]. Conversely, in patients with rosacea, serum vitamin D levels were found to be higher than those of the control groups [114]. Therefore, the pathogenesis of rosacea appears to differ somewhat from that of other chronic cutaneous inflammatory diseases.

Vitamin D is a secosteroid that is primarily involved in mineral homeostasis and bone metabolism [113]. In addition, vitamin D can act as an immune regulator for innate and adaptive immunity. Because vitamin D has many immunomodulatory activities, the regulation of several types of immune cells by vitamin D might have some clinical implications in determining the degrees of susceptibility to chronic inflammatory diseases [112,113,115].

Vitamin D can act as a potent inducer of LL-37. A Vitamin D responsive element (VDRE) has been identified in the human CAMP promoter [116]. Stimulation of VDRE by vitamin D binding to the vitamin D receptor (VDR) followed by heterodimerization with the retinoid acid receptor facilitates expression from the cathelicidin promoter in keratinocytes and monocytes [116]. In patients with fulminant rosacea, an association with a BsmI polymorphism in the *VDR* gene has been observed [117]. Specifically, a preponderance of the less active VDR allele 1 was detected, implying an association between VDR and the retinoid acid receptor pathway in rosacea [117].

In addition, vitamin D has been shown to increase the expression of *TLR2* and *KLK5* mRNA [118]. Therefore, vitamin D likely influences the TLR-2, KLK-5, and LL-37 associated pathway in rosacea and affects the subsequent proinflammatory cascades that can alter the immune system in these patients.

### 4.2. Dysregulation in Adaptive Immunity

#### 4.2.1. T Cell-Mediated Responses

A molecular characterization of inflammatory infiltrations in rosacea by Buhl et al. [14] demonstrated an increase of CD4^+^ over CD8^+^ T cells among the T cell population, and subsequent transcriptome data identified an upregulation of Th1 and Th17 polarizing gene sets [14]. In addition, an upregulation of IFN-γ and IL-17A in rosacea-affected skin was also identified [14]. IL-17 has been shown to induce angiogenesis through VEGF [94] and can also affect the expression of LL-37 in human epidermal keratinocytes. Therefore, Th17 cytokines might have some effect on the abnormal expression of LL-37 that is observed in rosacea [119].

The transcriptional analysis by Buhl et al. [14] also revealed the upregulation of mRNA for CXCL8, a major neutrophil chemotactic factor in rosacea. Similarly, other chemokines including CXCL1, CXCL2, CXCL5, and CXCL6 were also upregulated in patients with rosacea [14]. These chemokines exhibit angiogenic properties and are able to attract neutrophils and Th17 cells in rosacea [14]. The robust proinflammatory cytokines and chemokines in rosacea thus augment the infiltration of inflammatory cells and promote further immunes responses. In addition, the expression patterns of cytokines and chemokines provides additional evidence for their association with the Th1/Th17 pathways and rosacea.

To assess the level of regulatory T cells in rosacea, the mean percentage of CD4^+^CD25^+^ regulatory T cells was examined and determined to be higher in rosacea compared with lupus erythematosus [120]. This finding suggests that the infiltrating cells of rosacea better preserve their immunologic tolerance than those in other autoimmune disorders.

The erythroid differentiation regulator 1 (Erdr1), which is highly expressed and acts as a survival factor in various cells, was first discovered in murine and human erythroleukaemia cell lines that exhibited hemoglobin synthesis-inducing activity. Erdr1 is usually expressed in normal skin epithelium [121]; however, we recently demonstrated that epidermal Erdr1 expression was decreased in patients with rosacea compared to controls, whereas IL-18 was upregulated [102]. Consistent with this inverse correlation, we also observed that the Erdr1 was negatively regulated by IL-18 in rosacea [102]. IL-18 modulates the immune response by activating Th1-mediated responses [102] such as those observed in many other chronic inflammatory disorders including psoriasis and atopic and contact dermatitis.

In addition, Erdr1 has been shown to decrease ROS levels through suppressing heat shock protein 90 [122]. As UV radiation represents one of the triggering factors for rosacea, we suspected that recombinant Erdr1 might decrease the UV-induced oxidative stress in rosacea [102]. Accordingly, our recent study found that treatment with recombinant Erdr1 suppressed the expression of VEGF and reduced the angiogenesis in a murine rosacea model, implying its therapeutic possibilities for human patients with rosacea [102].

#### 4.2.2. B Cell-Mediated Responses

To date, in comparison to that of T cells, the role of B cells has been undervalued as a pathogenic factor in rosacea. However, approximately 10% to 20% of inflammatory cell infiltration in rosacea is composed of CD20+ B cells [40]. Furthermore, plasma cells are also observed in rosacea skin biopsy specimens [40] and infiltration with plasma cells along with profound antibodies has also been observed in rosacea [14]. In particular, antinuclear antibody titer has been frequently found to be elevated in patients with rosacea [123]. These findings suggest that the functions of B cell-mediated responses in rosacea warrant further consideration.

Fibrotic changes in the skin, which also can be manifested in phymatous rosacea, can be stimulated by B cells through the production of fibrogenic cytokines such as IL-6 and TGF-β via TLR [13,124]. In turn, TLR agonists stimulate the differentiation of plasma cells from B cells [125]. Furthermore, some antigen-specific antibody responses require TLR activation in B cells [125]. Although this mechanism has only been studied in a scleroderma model, further studies should be performed to determine whether fibrosis after TLR-associated B cell stimulation is also observed in rosacea models.

## 5. Neurovascular Dysregulation

Flushing and burning sensations in the skin are considered to represent a main clinical feature in rosacea and are regarded as being primarily caused by neurovascular dysregulation. In patients with rosacea, dilatations of the precapillary arterioles lead to flushing and erythema and dilatations of the postcapillary venules result in edema caused by protein leakage and the recruitment of leukocytes [1]. In addition, activated vascular endothelial cells express adhesion molecules and cytokine receptors, which aggravate the inflammation in rosacea [126]. Among these, VEGF is important for regulation of vascular permeability [127]. Expression of VEGF is increased in lesional skin of rosacea, and it may play a role in rosacea by leading erythema and angiogenesis [127].

Various neuropeptides and associated pathways involved in the vasodilation of rosacea have been proposed. For example, the expressions of neuromediators such as pituitary adenylate cyclase-activating polypeptide (PACAP), vasoactive intestinal peptide (VIP), adrenomedullin, calcitonin gene-related peptide (CGRP), and substance P were found to be enhanced in rosacea [105]. CGRP is involved in vasodilatation and substance P is important in the manifestation of edema in patients with rosacea via the neurokinin 1 receptors of the postcapillary venules [128,129]. Additional biologic actions of substance P in rosacea include mast cell degranulation, endothelial cell proliferation, and local axon reflex-associated vasodilatation [22].

Injection with PACAP, a neuropeptide responsible for edema and flushing in human skin, upregulates the expression of the mast cell protease, MMP-1, and MMP-9, which could further affect the pathway responsible for cleaving pro-protein hCAP18 into LL-37 [12,130]. Additionally, PACAP injection significantly increased the expression of proinflammatory cytokines such as TNF and CXCL2 [110]. These peptides might act collaboratively in the pathogenic mechanisms of rosacea by promoting inflammation and tissue damage [131]. Moreover, PACAP mediates vasodilation and the extravasation of erythrocytes, which might further affect neurogenic inflammation in rosacea [128].

Finally, TLR-2 is also expressed in sensory neurons. As the TLR-2 signaling pathways have the ability to mediate further neural dysregulation [95,132], the complex association between neural dysregulation and innate immunity in rosacea might therefore be explained by TLR-2. Thus, although the definitive roles of these mediators require further elucidation, their collective effects might underlie the vasodilatation and neurovascular dysfunction observed in rosacea.

## 6. Treatment Options for Rosacea

**Figure 2:**
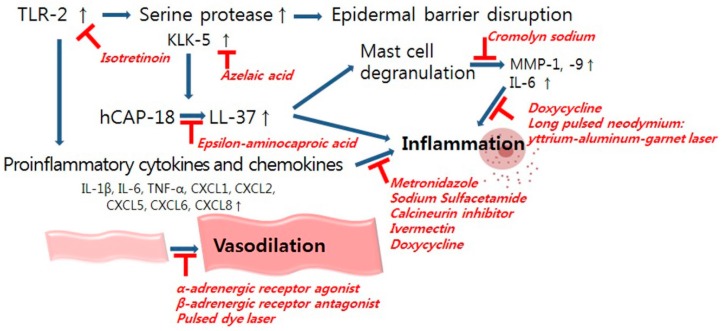
Primary pathogenic targets in rosacea with their respective treatment options. Blue lines indicate pathophysiological pathways involved in rosacea. Red lines illustrate major target pathways associated with the management options. Red words indicate management options in rosacea. Upward arrows indicate increased expression of certain protein.

Numerous treatment modalities are emerging in the treatment of rosacea, although avoidance of known triggering factors remains the first step toward relieving symptoms. Among the various therapeutic options, the determination of optimal treatment modalities depends on the clinical severity of the patient and the underlying mechanism of action of each treatment. Below we present the various treatment options of rosacea based on its main pathogenic targets and supposed molecular mechanisms (Figure 2).

### 6.1. Management of Inflammation

Doxycycline is well known to be an effective treatment for rosacea. Doxycycline inhibits the production and activity of MMP-9 [133] and also inhibits other MMPs, causing conformational changes and functional abnormalities [134]. Doxycycline has been further shown to indirectly inhibit the activation of KLK in vitro [135]. In addition, doxycycline was found to inhibit the NO synthase activity associated with vasodilatation, which might explain its anti-inflammatory properties in rosacea [136]. In particular, patients treated with a sub-antimicrobial-dose of doxycycline exhibited decreased inflammatory lesion counts and erythema scores [137]. Furthermore, a sub-antimicrobial-dose of doxycycline was recently shown to decrease the release of inflammatory cytokines and downregulate the production of ROS more effectively than a higher, antimicrobial dose in patients with rosacea [138].

The topical effects in rosacea of metronidazole, another well-known antibiotic, are similarly associated with its anti-inflammatory activity rather than its antimicrobial activity. Metronidazole works by reducing the generation of ROS and inactivating existing ROS, which decreases the release of further proinflammatory cytokines in rosacea [139]. It also prevents further tissue injury by suppressing release of cytokines from neutrophil [140]. 

Topical azelaic acid was shown to inhibit the expression of KLK-5 in cultured keratinocytes [141]. Similarly, after treatment with azelaic acid, the expression of KLK-5 and cathelicidin mRNA, factors associated with major rosacea pathophysiologic pathways as previously described, decreased in patients with rosacea as well [141]. Azelaic acid was shown to exert its anti-inflammatory properties by inhibiting the production of ROS and the UVB-induced upregulation of proinflammatory cytokines such as IL-1, IL-6, and TNF-α [142].

Although retinoids have not been approved for the treatment of rosacea, low-dose isotretinoin has been demonstrated to have a profound effect in papulopustular rosacea [143]. Isotretinoin was shown to downregulate TLR-2 expression and further regulate TLR-2-mediated abnormal innate immune responses in rosacea [144]. In addition, 13-*cis* retinoic acid leads to the atrophy of sebaceous glands, which might contribute to a reduction of demodex proliferation [143].

The effects of sodium sulfacetamide on rosacea are also mediated through its anti-inflammatory properties, although the precise underlying pathomechanisms have not been fully elucidated [145]. In addition, the combination of topical sodium sulfacetamide with sulfur yielded a decrease in the inflammatory lesion counts of rosacea. As sodium sulfacetamide exhibits anti-bacterial activity and sulfur exerts antidemodectic activity, its use might be beneficial toward improving the clinical manifestations of rosacea [142].

In comparison, the anti-parasitic agent ivermectin binds to the glutamate-gated chloride channels of different parasites including onchocerciasis, strongyloidiasis, pediculosis, and scabies [146]. It has been shown to reduce the inflammatory papules of rosacea, exhibiting its anti-inflammatory properties by inhibiting the NF-κB pathway, which decreases the production of proinflammatory cytokines, such as IL-1β and TNF-α [146,147].

The anti-inflammatory properties of calcineurin inhibitors have been exploited as well for the topical treatment of rosacea. Although the underlying mechanisms of topical calcineurin inhibitors in rosacea still need to be clarified, topical calcineurin inhibitors are thought to block T cell activation and thereby reduce the further release of proinflammatory cytokines in rosacea [148].

Furthermore, patients with erythematotelangiectatic rosacea showed reduced MMP activity after topical application of the mast cell inhibitor cromolyn sodium for 8 weeks. Because mast cells are major mediators of cathelicidin-associated cutaneous inflammation, a topical mast cell stabilizer to prevent the release of allergic/inflammation mediators might be useful in rosacea, although more large-scale studies are needed to confirm this association [110].

Similarly, the topical serine protease inhibitor epsilon-aminocaproic acid might be helpful in managing rosacea because it inhibits the production of active cathelicidin LL-37 from its inactive hCAL-18 form. In support of this conjecture, patient groups showed decreased erythema and papules clinically after treatment with serine protease inhibitors, along with reduced serine protease activity [149]. Therefore, serine protease inhibitors are considered an emerging therapy in rosacea.

Another widely used treatment modality in rosacea is intense pulsed light (IPL). The proposed mechanism for IPL is the remodeling of dermal collagen and vascular structure [150]. Recently, Lee et al. [151] found that light emitting diode irradiation using rosacea-like mouse model downregulated the mRNA expression of TLR-2, KLK-5, and LL-37, which are key factors in rosacea pathogenesis. Further clinical studies are necessary to prove the effectiveness of light emitting diode in patients with rosacea. In addition, a long-pulsed 1064-nm neodymium: yttrium-aluminum-garnet laser (LPND) was used to treat papulopustular rosacea, with good clinical efficacy in the patient group [152]. The use of LPND in a murine model of rosacea revealed that it reduced the expression of *MMP1* mRNA and increased the expression of TGF-β in hairless mice [153]. Therefore, LPND might be useful in treating rosacea by inducing the remodeling of dermal collagen.

### 6.2. Management of Vascular Dysregulation

The topical alpha adrenergic receptor agonists, brimonidine and oxymetazoline, act on the smooth muscles of the superficial and deep vascular plexus and mediate vasoconstriction around the dermal vessels. Although alpha adrenergic receptor agonists do not affect the capillaries because they do not contain smooth muscle, their action on the cutaneous arterioles reduces the erythema of patients with rosacea [154]. However, a recent study reported a paradoxical erythema reaction and allergic contact dermatitis after the use of brimonidine [155,156]. Therefore, further studies are required to demonstrate the prevalence of this phenomenon.

The use of systemic β-adrenergic receptor antagonist has also been proposed in rosacea for their vasoconstrictive properties on the smooth muscle of the dermal vasculature [157]. In particular, the nonselective β-adrenergic receptor blocker, carvedilol, has been shown to exhibit profound effects on improving rosacea-associated erythema with decreased side effects in severe refractory cases [157].

In addition, a 585-nm pulsed dye laser (PDL) has also been widely used to manage rosacea-associated erythema and telangiectasia [158,159]. In one study, two patients with rosacea were treated with a single session of 585 nm PDL [160], which led to a decrease in the immunoreactivity of substance P-positive nerve fibers. However, expression of the neuropeptides CGRP and VIP, which are also involved in the vascular pathophysiology of rosacea, did not decrease after treatment. Therefore, substance P is considered to selectively represent a potential molecular target for PDL therapy in rosacea.

## 7. Conclusions

**Figure 3:**
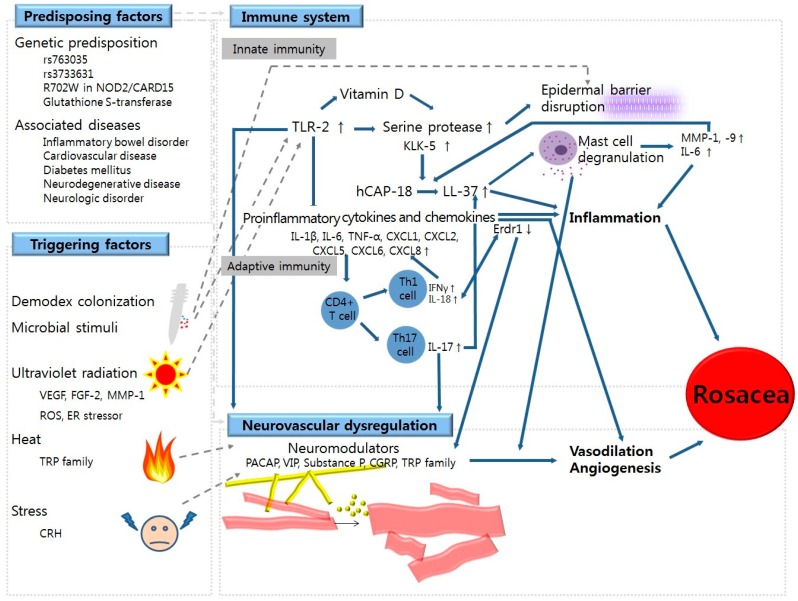
Representation of proposed mechanisms for rosacea. Predisposing factors are associated with dysregulated immune responses and neurovascular dysregulation. Among the triggering factors, demodex proliferation directly disrupts the epidermal barrier. Demodex and ultraviolet radiation can cause the elevated expression of TLR-2. TLR-2 regulates the release of KLK-5, which disrupts the epidermal barrier and activates the cleavage of hCAP-18 into LL-37. LL-37 stimulates tissue inflammation, vasodilation, and angiogenesis in rosacea. LL-37 also facilitates the degranulation of mast cells, which further enhances the expression of MMP-1, MMP-9, and IL-6. Furthermore, Th1 and Th17-mediated immune reactions affect inflammation in rosacea. In addition, Erdr1 is related to the upregulation of IL-18 and angiogenesis. Heat and stress can induce the release of various neuromodulators, such as PACAP, VIP, substance P, CGRP, and the TRP family. These cause neurovascular dysregulation, which consequently results in inflammation, vasodilation, and angiogenesis in rosacea. Blue solid lines indicate pathophysiological pathways involved in rosacea. Dashed lines denote the proposed mechanisms for triggering factors and predisposing factors in rosacea. Upward arrows indicate increased expression of certain protein. Abbreviation: TLR, Toll-like receptor; KLK-5, Kallikrein-5; MMP, Matrix metalloproteinase; PACAP, Pituitary adenylate cyclase-activating polypeptide; VIP, Vasoactive intestinal peptide; CGRP, Calcitonin gene-related peptide; TRP, Transient receptor potential, VEGF, Vascular endothelial growth factor; FGF, Fibroblast growth factor; ROS, Reactive oxygen species.

Many investigations have addressed the pathophysiology of rosacea. Although the genetic predisposition to rosacea has not yet been widely studied, it is clear that some genetic association does exist in rosacea, potentially to a degree equivalent to that of environmental influences. In addition, various triggering factors, such as demodex colonization, microbial stimuli, UV radiation, heat, and stress are implicated in the development or worsening of rosacea. Abnormal functioning of TLR-2, KLK-5, and cathelicidin or vitamin D response along with epidermal barrier disruption may contribute to the dysregulation of innate immunity and augment the inflammatory cascade in rosacea. Furthermore, Th1 and Th17 cell-mediated immune responses might affect the adaptive immunity of patients with rosacea as well. Vascular alteration and neuromodulators, which consequently lead to inflammation, vasodilatation, and angiogenesis, also constitute the complex network of rosacea (Figure 3). However, the precise cause of rosacea remains unclear and the etiologies of rosacea appear to be multifactorial. Further studies regarding additional predisposing factors for rosacea are thus warranted.

Previous treatment options for rosacea have been based on the results of clinical trials and have been mainly reliant on modes of action related to symptomatic and anti-inflammatory effects. Recently emerging treatment options such as mast cell stabilizers and protease inhibitors also act against the inflammatory cascade of rosacea. As demodex appears to represent an important contributory factor for rosacea induction, further molecular biological investigations are required to identify effective means for increasing the human defense mechanisms against demodex mite proliferation.

In summary, a better understanding the molecular mechanisms of rosacea might promote a clear comprehension of the complex linkages in its pathophysiology. Further studies based on this review might thereby provide a bridge toward more targeted and individualized treatment options for patients with rosacea.

## Abbreviations

AMPs	Antimicrobial peptides
BRNL2	Butyrophilin-like 2
CAMP	Cathelicidin antimicrobial peptide
CGRP	Calcitonin gene-related peptide
CRH	Corticotrophin- releasing hormone
CRH-R	Cortisol releasing hormone receptor
ER	Endoplasmic reticulum
Erdr1	Erythroid differentiation regulator 1
GST	Glutathione S-transferase
GWAS	Genome-wide association study
HLA	Human leukocyte antigen
IL	Interleukin
IPL	Intense pulsed light
KLK	Kallikrein
LPND	Long pulsed 1064-nm neodymium: yttrium-aluminum-garnet laser
MMP	Matrix metalloproteinase
MyD88	Myeloid differentiation factor 88
NF-κB	Nuclear factor κ-light-chain-enhancers of activated B cells
NLRP3	Nucleotide-binding oligomerization domain-like receptor family, pyrin domain containing 3
NO	Nitric oxid
PACAP	Pituitary adenylate cyclase-activating polypeptide
PAR-2	Protease-activated receptor 2
PDL	Pulsed dye laser
PERK	Protein kinase R-like endoplasmic reticulum kinase
PRRs	Pattern recognition receptors
ROS	Reactive oxygen species
SNP	Single-nucleotide polymorphism
SPINK	Serine protease inhibitor of Kazal-type
SSNA	skin sympathetic nerve activity
TLR	Toll-like receptor
TNF	Tumor necrosis factor
TRPV1	Transient receptor potential cation channel subfamily V member 1
UV	Ultraviolet
VEGF	Vascular endothelial growth factor
VDR	Vitamin D receptor
VEGF	Vascular endothelial growth factor
VIP	Vasoactive intestinal peptide
